# Supplementary material for: The impact of spectral basis set composition on estimated levels of cingulate glutamate and its associations with different personality traits
Source: BMC Psychiatry. 2024 Apr 25;24:320. doi: 10.1186/s12888-024-05646-x (PMC11044602; doi:10.1186/s12888-024-05646-x)
Supplement: Supplementary file 1 — Supplementary Material 1 [file 12888_2024_5646_MOESM1_ESM.pdf]

Supplementary Materials

The impact of spectral basis set composition on estimated levels of cingulate glutamate and its associations with different personality traits

Verena Demler, Elisabeth F. Sterner, Martin Wilson, Claus Zimmer, Franziska Knolle

1 Introduction:

Table S1:

Metabolites	A	B	C	D	E	F	G	H	I
Ala			X	X	X	X	X	X	X
Asc			X		X				
Asp	X	X	X	X	X	X	X	X	X
Ch	X								
Cr	X	X	X	X	X	X	X	X	X
GABA	X	X	X	X	X	X	X	X	X
Glc		X	X	X	X	X	X	X	X
Gln	X	X	X	X	X	X	X	X	X
Glu	X	X	X	X	X	X	X	X	X
Gly					X	X		X	
GSH		X	X	X	X	X		X	X
GPC		X	X	X	X	X	X	X	X
Lac			X	X	X	X	X	X	X
mI	X	X	X	X	X	X	X	X	X
NAA	X	X	X	X	X	X	X	X	X
NAAG	X	X	X	X	X	X	X	X	X
PCh		X	X		X	X	X	X	X
PCr	X	X	X	X		X		X	
PE			X		X	X		X	
sI	X	X	X	X	X	X	X	X	X
Tau	X	X	X	X	X	X	X	X	X
GA							X		

Note: Composition of basis sets for the study of A) Cheng et al. (1), B) Maddock et al. (2), C) Reid et al. (3), D) LCModel Manual (4), E) Kozhuharova et al. 2021(5), F) Leptourgos et al. 2023 (6), G) Ford et al. 2017 (7), H) Shukla et al. 2019 (8) and I) Rowland et al. 2013 (9); Ala, alanine; Asc, ascorbate; Asp, aspartate; Cr, creatine; Glc, glucose; Glu, glutamate; Gln, glutamine; Gly, glycine; GSH, glutathione; GPC, glycerophosphocholine; Lac, lactate; mI, myo-Inositol; NAA, N-acetylaspartate; NAAG, N-acetylaspartylglutamate; PCh, phosphocholine; PCr, phosphocreatine; PE, phosphoroylethanolamine; sI, scyllo-Inositol; Tau, taurine; GA, guanidinoacetate. Grey background indicates example basis sets chosen for analysis in this study.

2 Methods

2.1 Participants

Inclusion criteria: native German speaker; right-handed; no diagnosis of schizophrenia, psychosis or autism, or any neurological disease or injury; not currently taking any psychoactive medication within at least the past six weeks and no contraindications for MRI scanning.

Subjects: Out of the participants, three had been previously diagnosed with depression, two had prior eating disorders, one was diagnosed with schizoid personality disorder along with adjustment disorder, and another reported having post-traumatic stress disorder and narcissistic personality disorder. However, only one of the participants was consistently taking antipsychotic medication (fluoxetine, atomoxetine) for attention deficit disorder.

Details of demographic data and symptom scores are shown in Table S2 and have been previously published in (10).

Table S2: Demographic data and clinical scores.

	Female (n = 26)	Male (n = 27)	P-value[a]	W
Age	23.31 (3.54)	23.93 (4.21)	0.7266	331
SPQ: Positive-like Symptoms (/132)	35.96 (23.54)	28.70 (25.20)	0.1846	426
SPQ: Negative-like Symptoms (/100)	37.85 (15.57)	34.33 (20.49)	0.3454	404
SPQ: Disorganized Traits (/64)	23.19 (14.27)	21.22 (13.12)	0.7285	371
AQ: Total Score (/50)	21.65 (6.99)	20.96 (7.65)	0.7017	373

Note: Values are mean (SD)

SPQ, Schizotypal Personality Questionnaire; AQ, Autism Spectrum Quotient

[a] Wilcoxon rank sum test

2.2 ^{1}H-MRS Processing

See Figure S1 for an overview of our processing, modelling and basis set workflow.

2.3 Correlations of metabolite concentration estimates with age

To offer an additional objective marker for the reliability of each method, we explored the relationship between the different metabolite concentrations and age, separated by sex. We, therefore, calculated Spearman's rank correlation coefficients of Glu/tCr, absolute glu and glx with age. The correlation analyses were performed in R using the rstatix package (11) (version 0.7.1). The visualisation of the scatterplots and the statistics in the plots were created with the ggpubr package (12) (version 0.5.0).

3 Results and Discussion

3.1 Spectral quality

We applied the paired Wilcoxon signed-rank test for multiple pairwise comparisons of the CRLB and the multiple pairwise paired t-tests for the SNR between basis sets. P-values were adjusted using the

Bonferroni multiple-testing correction method. An adjusted p-value of less than 0.05 was considered significant. Results are presented in **Table S3 - 7**.

Table S3: Summary spectral quality parameter distributions of the basis sets

**Quality Parameter**		**Rowland**	**LCModel**	**Maddock**	**Reid**	**Kozhuha.**	**Group comparisons**	
Analysis method	**n**	**Mean ± SD**	**Mean ± SD**	**Mean ± SD**	**Mean ± SD**	**Mean ± SD**	**Statistic**	**P-value**
**Glu CRLB (%)**								
Spant + ABfit	53	4.04 ± 0.88	3.79 ± 0.74	3.87 ± 0.68	3.62± 0.86	3.83 ± 0.78	X^2_4 = 40.33[a]	**<0.0001**
Osprey + LCM	53	5.32 ± 0.58	5.25 ± 0.52	5.25 ± 0.52	5.09 ± 0.66	5.25 ± 0.55	X^2_4 = 17.45[a]	**0.0016**
**Glx CRLB (%)**								
Spant + ABfit	53	1.83 ± 0.67	1.77 ± 0.51	1.89 ± 0.47	1.81 ± 0.59	1.62 ± 0.56	X^2_4 = 12.40[a]	**0.0146**
Osprey + LCM	53	5.17 ± 0.67	5.21 ± 0.50	5.23 ± 0.51	4.98 ± 0.69	4.93 ± 0.76	X^2_4 = 27.47[a]	**<0.0001**
**SNR**								
Spant + ABfit	53	149.18 ± 18.01	148.02 ± 18.33	147.88 ± 18.20	149.23 ± 18.36	149.89 ± 18.05	$F_{1.68, 87.49}$ = 31.03[b]	**<0.0001**
Osprey + LCM	53	101.06 ± 13.88	101.06 ± 13.88	101.06 ± 13.88	101.06 ± 13.88	101.06 ± 13.88	NaN[b]	NaN
**FWHM (ppm)**								
Spant + ABfit	53	0.042 ± 0.01	0.041 ± 0.01	0.041 ± 0.01	0.041 ± 0.01	0.042 ± 0.01	X^2_4 = 150.7[a]	**<0.0001**

**FWHM (Hz)**								
Osprey + LCM	53	6.18 ± 0.44	6.18 ± 0.44	6.18 ± 0.44	6.18 ± 0.44	6.18 ± 0.44	NaN [a]	NaN

Note: Kozhuha.., Kozhuharova; SNR, Signal-to-Noise Ratio; CRLB, Cramer-Rao lower bounds; FWHM, full width at half maximum, in Spant: measurement for the tNAA (NAA + NAAG) linewidth in Osprey: measurement for the water linewidth

[a] Calculated using the Friedman test

[b] Calculated using the repeated measures ANOVA

Table S4: Pairwise comparisons for the SNR

		spant+ABfit		*Osprey+LCM*	
**group1**	**group2**	**statistic**	**P-value adj.**	**statistic**	**P-value adj.**
Rowland	LCModel	5.20	**<0.0001**	NA	NA
Rowland	Maddock	5.57	**<0.0001**	NA	NA
Rowland	Reid	-0.24	1	NA	NA
Rowland	Kozhuharova	-5.03	**<0.0001**	NA	NA
LCModel	Maddock	1.88	0.662	NA	NA
LCModel	Reid	-8.84	**<0.0001**	NA	NA
LCModel	Kozhuharova	-6.76	**<0.0001**	NA	NA
Maddock	Reid	-9.36	**<0.0001**	NA	NA
Maddock	Kozhuharova	-6.84	**<0.0001**	NA	NA
Reid	Kozhuharova	-2.27	0.272	NA	NA

Note: Multiple pairwise paired t-tests for the SNR of spant+ABfit and Osprey+LCM analyses; SNR, Signal-to-Noise Ratio; P-value adj.; adjusted p-value using the Bonferroni multiple-testing correction.

Table S5: Pairwise comparisons for Glu CRLB

		spant+ABfit		*Osprey+LCM*	
group1	group2	statistic	P-value adj.	statistic	P-value adj.
Rowland	LCModel	352	0.201	38.5	1
Rowland	Maddock	285	0.971	38.5	1
Rowland	Reid	409	**0.005**	97.5	**0.014**
Rowland	Kozhuharova	85.5	0.277	17.5	1
LCModel	Maddock	3.5	1	0	NA
LCModel	Reid	66	0.226	49.5	0.121
LCModel	Kozhuharova	138.5	1	18	1
Maddock	Reid	85	**0.034**	49.5	0.121
Maddock	Kozhuharova	188	1	18	1
Reid	Kozhuharova	94	0.459	13	0.212

Note: Wilcoxon signed-rank test pairwise comparisons for the glutamate CRLB of spant+ABfit and Osprey+LCM analyses; CRLB, Cramer-Rao lower bounds; P-value adj., adjusted p-value using the Bonferroni multiple-testing correction.

Table S6: Pairwise comparisons for Glx CRLB

		spant+ABfit		*Osprey+LCM*	
group1	group2	statistic	P-value adj.	statistic	P-value adj.
Rowland	LCModel	132	1	45	1
Rowland	Maddock	120	1	35	1
Rowland	Reid	121	1	110.5	0.135
Rowland	Kozhuharova	79	0.132	98	**0.018**
LCModel	Maddock	4.5	0.411	0	1
LCModel	Reid	13.5	1	119	**0.03**

LCModel	Kozhuharova	123.5	0.628	228	**0.019**
Maddock	Reid	38.5	1	135	**0.018**
Maddock	Kozhuharova	178.5	**0.019**	218.5	**0.007**
Reid	Kozhuharova	142.5	0.349	110	1

Note: Wilcoxon signed-rank test pairwise comparisons for the Glx CRLB of spant+ABfit and Osprey+LCM analyses; CRLB, Cramer-Rao lower bounds; P-value adj., adjusted p-value using the Bonferroni multiple-testing correction.

Table S7: FWHM

		spant+ABfit		*Osprey+LCM*	
**group1**	**group2**	**statistic**	**P-value adj.**	**statistic**	**P-value adj.**
Rowland	LCModel	1428	**<0.0001**	NA	NA
Rowland	Maddock	1428	**<0.0001**	NA	NA
Rowland	Reid	1430	**<0.0001**	NA	NA
Rowland	Kozhuharova	966	0.269	NA	NA
LCModel	Maddock	723	1	NA	NA
LCModel	Reid	994	0.139	NA	NA
LCModel	Kozhuharova	4	**<0.0001**	NA	NA
Maddock	Reid	819	1	NA	NA
Maddock	Kozhuharova	1	**<0.0001**	NA	NA
Reid	Kozhuharova	0	**<0.0001**	NA	NA

Note: Wilcoxon signed-rank test pairwise comparisons for the FWHM of tNAA for spant+ABfit and of water for Osprey+LCM analyses; FWHM, full width at half maximum; P-value adj., adjusted p-value using the Bonferroni multiple-testing correction.

3.2 Group differences between the metabolite concentration estimates

Comparisons between the estimates for Glu/tCr, Glu, and Glx are summarized in **Figure 4**, results are presented in **Table S8-S11.**

Table 8: Differences between the metabolite concentration distributions of the basis sets

Metabolite		Rowland	LCModel	Maddock	Reid	Kozhuha.	Group comparisons	
Analysis method	n	Mean ± SD	Mean ± SD	Mean ± SD	Mean ± SD	Mean ± SD	Statistic	P-value
**Glu/tCR**								
Spant + ABfit	53	1.71 ± 0.22	1.49 ± 0.22	1.46 ± 0.22	1.46 ± 0.27	1.76 ± 0.22	$X^2_4 = 72.15$	<0.0001
Osprey + LCM	53	1.69 ± 0.11	1.56 ± 0.10	1.55 ± 0.10	1.53 ± 0.11	1.68 ± 0.10	$X^2_4 = 166.88$	<0.0001
**Glu**								
Spant + ABfit	53	20.53 ± 2.87	18.18 ± 2.95	17.72 ± 2.93	18.01 ± 3.54	21.48 ± 2.86	$X^2_4 = 79.98$	<0.0001
Osprey + LCM	53	30.21 ± 1.93	28.12 ± 1.91	28.01 ± 1.90	28.13 ± 1.89	30.13 ± 1.81	$X^2_4 = 151.02$	<0.0001
**Glx**								
Spant + ABfit	53	24.28 ± 3.34	21.28 ± 3.23	20.81 ± 3.21	21.37 ± 4.01	26.39 ± 4.01	$X^2_4 = 89.51$	<0.0001
Osprey + LCM	53	36.56 ± 2.31	33.37 ± 2.07	33.28 ± 2.06	33.20 ± 216	36.50 ± 2.17	$X^2_4 = 149.69$	<0.0001

Note: Statistics are calculated using the Friedman test; all values were rounded to two decimal places. For Glu/tCr the glutamate concentration is referenced to tCr, except for Rowland and Kozhuharova

(Glu/Cr). The absolute Glu and Glx values are given in mol/kg. Kozhuha., Kozhuharova; Glu, glutamate; tCr, total creatin (creatin + phosphocreatine); Glx, glutamate + glutamine

Table S9: Pairwise comparisons for Glu/tCr

		spant+ABfit		Osprey+LCM	
**group1**	**group2**	**statistic**	**P-value adj.**	**statistic**	**P-value adj.**
Rowland	LCModel	1309	<0.0001	1428	<0.0001
Rowland	Maddock	1329	<0.0001	1429	<0.0001
Rowland	Reid	1292	<0.0001	1428	<0.0001
Rowland	Kozhuharova	491	0.474	1224	<0.0001
LCModel	Maddock	936	0.515	1406	<0.0001
LCModel	Reid	977	0.209	1256	<0.0001
LCModel	Kozhuharova	53	<0.0001	10	<0.0001
Maddock	Reid	777	1	1172	0.0005
Maddock	Kozhuharova	59	<0.0001	4	<0.0001
Reid	Kozhuharova	98	<0.0001	8	<0.0001

Note: Wilcoxon signed-rank test pairwise comparisons for Glu/tCr of spant+ABfit and Osprey+LCM analyses; Glu, glutamate; tCr, total creatin (creatin + phosphocreatine); P-value adj.; adjusted p-value using the Bonferroni multiple-testing correction.

Table S10: Pairwise comparisons for Glu

		spant+Abfit		Osprey+LCM	
**group1**	**group2**	**statistic**	**P-value adj.**	**statistic**	**P-value adj.**
Rowland	LCModel	1268	<0.0001	1426	<0.0001

Rowland	Maddock	1303	<0.0001	1429	<0.0001
Rowland	Reid	1186	0.0003	1420	<0.0001
Rowland	Kozhuharova	277	0.001	936	0.515
LCModel	Maddock	1055	0.027	1370	<0.0001
LCModel	Reid	873	1	719	1
LCModel	Kozhuharova	32	<0.0001	5	<0.0001
Maddock	Reid	598	1	551	1
Maddock	Kozhuharova	34	<0.0001	2	<0.0001
Reid	Kozhuharova	128	<0.0001	19	<0.0001

Note: Wilcoxon signed-rank test pairwise comparisons for absolute Glu values of spant+ABfit and Osprey+LCM analyses; Glu, glutamate; P-value adj.; adjusted p-value using the Bonferroni multiple-testing correction.

Table S11: Pairwise comparisons for Glx

		spant+Abfit		Osprey+LCM	
**group1**	**group2**	**statistic**	**P-value adj.**	**statistic**	**P-value adj.**
Rowland	LCModel	1295	<0.0001	1429	<0.0001
Rowland	Maddock	1324	<0.0001	1429	<0.0001
Rowland	Reid	1166	0.0006	1424	<0.0001
Rowland	Kozhuharova	118	<0.0001	802	1
LCModel	Maddock	1030	0.054	1232	<0.0001
LCModel	Reid	748	1	940	0.474
LCModel	Kozhuharova	9	<0.0001	1	<0.0001
Maddock	Reid	536	1	855	1
Maddock	Kozhuharova	8	<0.0001	0	<0.0001

Reid	Kozhuharova	118	**<0.0001**	5	**<0.0001**

Note: Wilcoxon signed-rank test pairwise comparisons for absolute Glx values of spant+ABfit and Osprey+LCM analyses; Glx, glutamate+glutamine; P-value adj.; adjusted p-value using the Bonferroni multiple-testing correction.

3.3 Group differences between the metabolite concentration estimates adding PCr

Comparisons between the estimates for Glx are summarized in **Figure S2A**. Adding PCr to the basis sets leads to a decrease in the Glx concentrations for both the Rowland(9) and the Kozhuharova(5) basis sets as well as in both toolboxes. Estimates were consistently lower for the analysis in spant+ABfit compared to the ones analyzed with Osprey+LCM. Necessary to note is also that the Rowland+PCr and LCModel basis sets are now identical, which can be nicely seen in the boxplots.

3.4 Correlations of each metabolite estimate between the basis sets

As already mentioned in the manuscript, the correlations between metabolite (Glu/tCr, absolute Glu, Glx) and basis set per toolbox revealed that the metabolite concentrations between the basis sets had higher correlations using Ospreys LCM integration than spant+ABfit (see Figure S3). For Osprey+LCM, we found strong correlations between all basis sets (r>0.75). Comparing the Rowland (9) and Kozhuharova (5) basis sets with added PCr to the ones without, the “new” basis sets show higher correlations with the three visually best-fitting basis sets (LCModel Manual (4), Maddock et al. (2), and Reid et al. (3); **Figure 3 B, C, and D**) with Spearman correlation coefficients between 0.95-1 for Osprey+LCM and 0.87-0.97 for spant+ABfit, the three visually best-fitting basis sets (LCModel Manual (4), Maddock et al. (2), and Reid et al. (3); **Figure 3 B, C, and D**) showed the best results with spearman correlation coefficients between 0.93-1 for Osprey+LCM and 0.88-0.94 for spant+ABfit (**Figure S3**). Correlation strength was classified according to Akoglu (13).

3.5 Fitting parameters influence the personality traits - glutamate correlations

Finally, we also analyzed Spearman's rank correlations for clinical scores with the extracted concentration scores for the two basis sets with added PCr. The correlations without additionally added PCr can be found in Figure S4. Overall, the results in Osprey+LCM and ant+ABfit displayed now a more homogenous pattern regarding their correlations. Using the Rowland (9)and Kozhuharova(5) basis set with PCr instead of the ones without this metabolite, the maximum difference between the correlation coefficients of Glx and disorganized traits in Osprey+LCM decreased from 0.18 to 0.04. The same can be seen in spant+ABfit where the maximal difference was changing from 0.42 to 0.16 for this correlation. However, the difference between the toolboxes was still smaller than the difference across the different basis sets and reached a maximum of 0.11, again for the correlation of Glx and disorganized traits. The correlations are shown in **Figure S2 B and C.**

3.6 Correlations of metabolite concentration estimates with age

We could not find any significant correlations between metabolite concentrations and age. Looking at Spearman's rank correlations for Glu/tCr, Glu and Glx with age separated by sex, we also found no significant results (Figure S5 and S6).

These results do not represent the finding of an age-related decrease in glutamate in adolescence and young adulthood, which has been reported in multiple studies across various regions (14–16). One reason for not detecting this effect might be that the age range in our dataset is too narrow.

Furthermore, we can see visual differences between the sexes in both toolboxes regarding the correlation of the metabolite concentrations with age. Whereas in spant+ABfit the correlations between the metabolite concentrations and age tend to be in the opposite direction for female and male participants, Osprey+LCM showed an almost parallel trend. For Osprey+LCM, the glutamate concentrations seem to be overall higher in males than in females. Changes in the glutamate concentration dependent on the sex have also been observed in previous studies (15,17).

4 Analysis with Gln

4.1 Quality assessment of Gln

Table S12 shows the mean values for the CRLB and the metabolite concentration of Gln for each basis set. Compared to the CRLBs of Glu and Glx, the mean values for Gln are around 15% higher and lie between 15.92% for the Reid basis set and 20.51% for the Rowland basis set. As the recommended CRLB exclusion cut-off lies at 20%, 31 participants in our study need to be excluded. As an information for future studies, the analysis of Gln with the reduced number of participants are presented here in the supplements.

*Table **S12**: Quality and Gln concentration*

Parameter		Rowland	LCModel	Maddock	Reid	Kozhuharova
Analysis method	n	Mean ± SD	Mean ± SD	Mean ± SD	Mean ± SD	Mean ± SD
**CRLB of Gln (%) before exclusion of participants**						
Spant + ABfit	53	20.37 ± 7.98	17.41 ± 7.87	17.79 ± 7.99	15.92 ± 5.37	17.73 ± 6.35
Osprey + LCM	53	20.51 ± 3.56	18.77 ± 4.04	18.7 ± 4.04	18.96 ± 4.42	20.4 ± 3.64
n exclusions (Spant/Osprey)		24/21	7/14	8/14	8/13	12/25
**After exclusion of 31 participants:**						
**CRLB of Gln (%)**						
Spant + ABfit	22	15.54 ± 1.89	12.71 ± 2.37	13.01 ± 2.39	12.26 ± 2.2	13.98 ± 1.95
Osprey + LCM	22	17.73 ± 1.28	15.5 ± 1.54	15.41 ± 1.62	15.55 ± 1.65	17.32 ± 1.09
**Gln in mol/kg**						
Spant + ABfit	22	4.35 ± 0.97	3.56 ± 0.58	3.56 ± 0.59	3.89 ± 1.22	5.72 ± 1.89
Osprey + LCM	22	8.42 ± 0.72	7.25 ± 0.72	7.26 ± 0.72	7.11 ± 0.8	8.56 ± 0.62

Note: n exclusions (Spant/Osprey), number of exclusions in Spant+ABfit and Osprey+LCM; Gln, glutamine

4.2 Group differences between Gln

Comparisons between the estimates for Gln for each analysis method are summarized in Figure S7A. Similar to the other metabolites estimates (Glu/tCr, Glu, Glx) we found a higher heterogeneity of Gln in spant+ABfit compared to Osprey+LCM based on the visualization of the individual data points and their connection over the different basis sets.

4.3 Fitting parameters influence the personality trait-glutamate correlations for Gln

Finally, we analyzed Spearman's rank correlations for clinical scores with Gln of the different basis sets (Figure S7B). Overall, the results in Osprey+LCM displayed a more homogenous pattern regarding the tendency of their non-significant correlations with a maximum difference of 0.2 between the coefficient scores for the correlation of Gln with disorganized traits. Whereas in spant+ABfit the correlations coefficients showed greater variability ranging from positive to negative values, with a maximal difference of 0.86 for the same correlation, which even has been significant for LCModel (4) ($r=0.55$, $p=0.006$), Maddock (2) ($r=0.52$, $p=0.013$) and Kozhuharova (5) ($r=-0.31$, $p=0.047$). These results are comparable to the effects shown with the other metabolite estimates (Glu/tCr, Glu, Glx), but show an even higher inhomogeneity (see Figure S5 and S6).

4.4 Correlations of Gln between the basis sets and toolboxes

Correlations between Gln and basis set per toolbox reveal also similar results compared to the other metabolite concentrations (Glu/tCr, Glu, Glx): between the basis sets Gln correlated higher using Ospreys LCM integration than using spant+ABfit. For Osprey+LCM, we found strong correlations between all basis sets ($r>0.75$). The quantification results in spant+ABfit showed a much higher variability, especially with the basis set of Kozhuharova et al. (5). Between the toolboxes, the correlations were weak to high, whereby the correlations have been generally higher for Gln than for the other basis sets, excluding Kozhuharova et al. (5). Correlation strength was classified according to Akoglu (13).

5 Figures

Figure S1: Processing, modeling, and basisset workflow

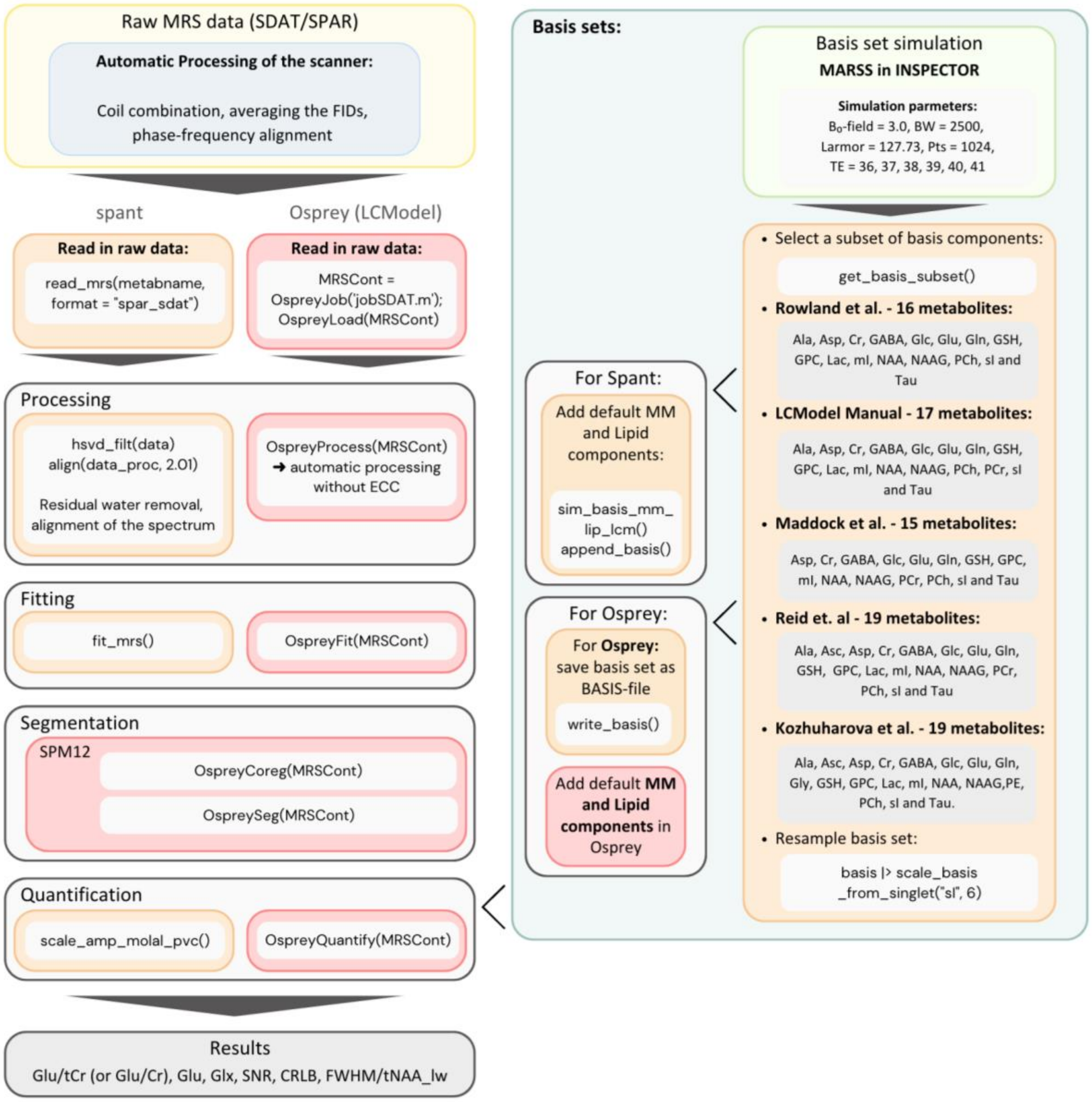

Note: Processing, modeling, and basisset workflow, summarizing the differences between the applied algorithms and basis sets; colors indicate the toolboxes used: orange, Spant; red, Osprey; green, MARSS in INSPECTOR; MRS, Magnetic resonance spectroscopy; ECC, Eddy current correction; MM, Macromolecules; Ala, alanine; Asc, ascorbate; Asp, aspartate; Cr, creatine; Glc, glucose; Glu, glutamate; Gln, glutamine; Gly, glycine; GSH, glutathione; GPC, glycerophosphocholine; Lac, lactate; mI, myo-Inositol; NAA, N-acetylaspartate; NAAG, N-acetylaspartylglutamate; PCh, phosphocholine; PCr, phosphocreatine; PE, phosphoroylethanolamine; sI, scyllo-Inositol; Tau, taurine; SNR, Signal-to-Noise

Ratio; CRLB, Cramer-Rao lower bounds; FWHM, full width at half maximum, measurement for the water linewidth; tNAA_lw, measurement for the tNAA (NAA + NAAG) linewidth

Figure S2: Basisets + PCr: Group comparisons for Glx in the ACC and Correlations between the metabolite concentrations and clinical scores

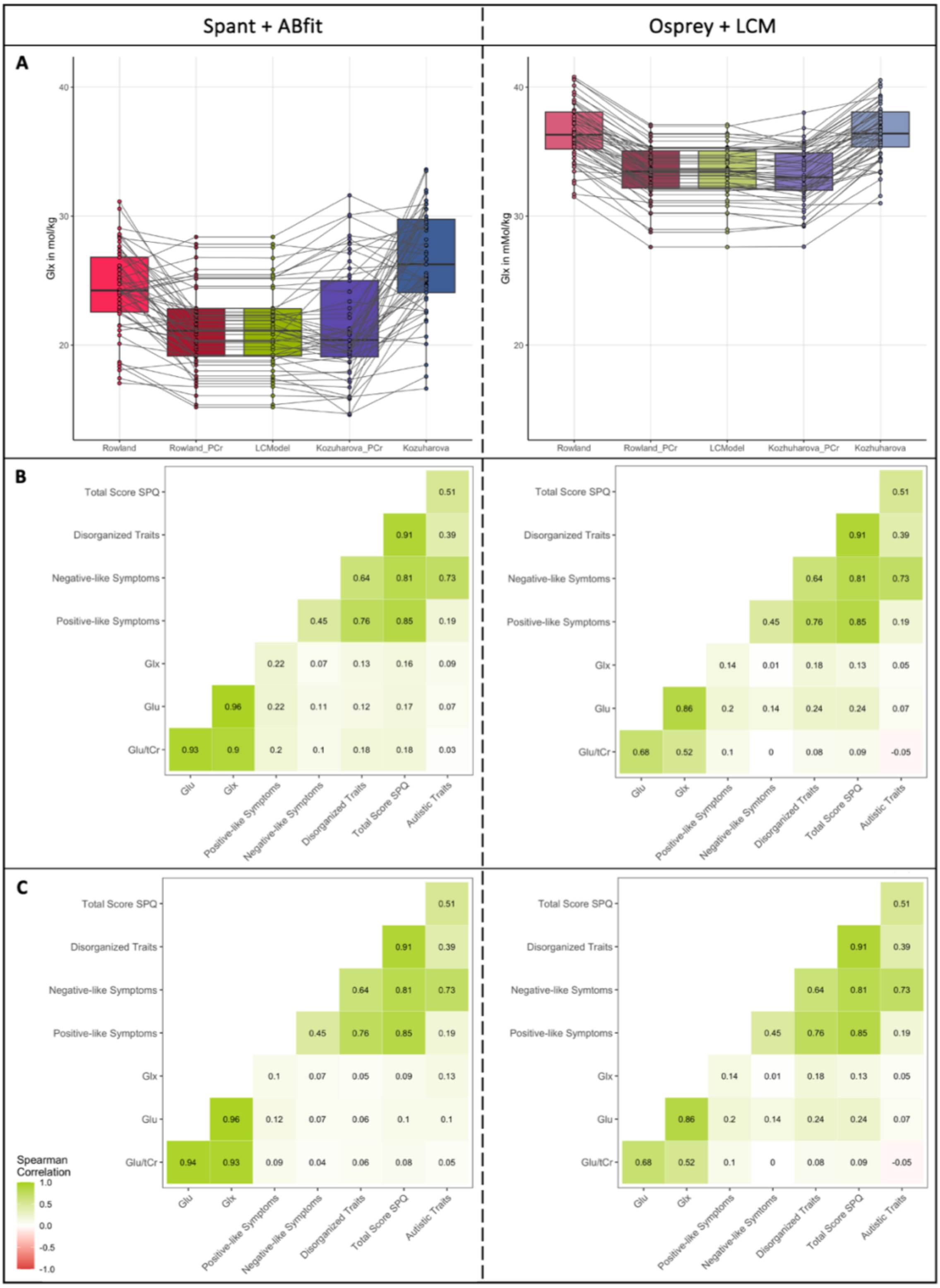

Note: A) Group comparisons between the basis sets (Rowland (9)*, Rowland + PCr, LCModel* (4)*, Kozhuharova +PCr, Kozhuharova* (5) *for absolute Glx values; Spearman correlations between Glu/tCr (Glu/Cr for Rowland and Kozhuharova), absolute Glu, Glx, and the subclinical traits for the basis sets B) Rowland + PCr and C)* (5)*; the left side always shows the results for spant+ABfit and the right side for Osprey+LCM. The psychotic-like traits are separated into the subscores: positive-like symptoms, negative-like symptoms, and disorganized traits.*

Figure S3: Basis sets + PCr tool box intercorrelations

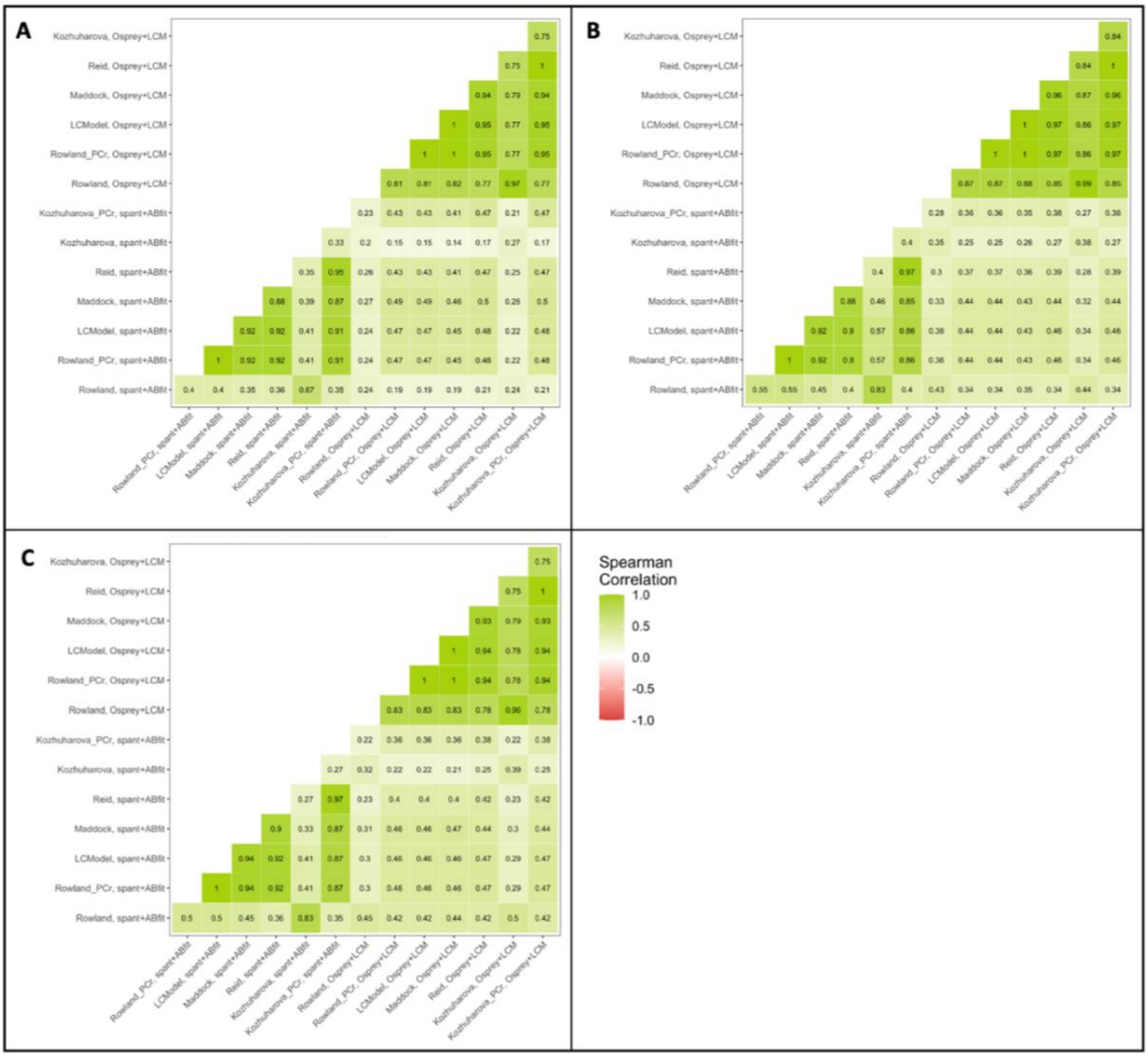

Note: Intercorrelations between the basis sets (Rowland (9)*, Rowland+PCr, LCModel* (4)*, Maddock* (2)*, Reid* (3)*, Kozhuharova* (5) *and Kozhuharova+PCr) and toolboxes (spant+ABfit; Osprey+LCM) for A) Glu/tCr (Glu/Cr for Rowland and Kozhuharova), B) Glu, C) Glx*

Figure S4: Correlations between the metabolite concentrations and clinical scores

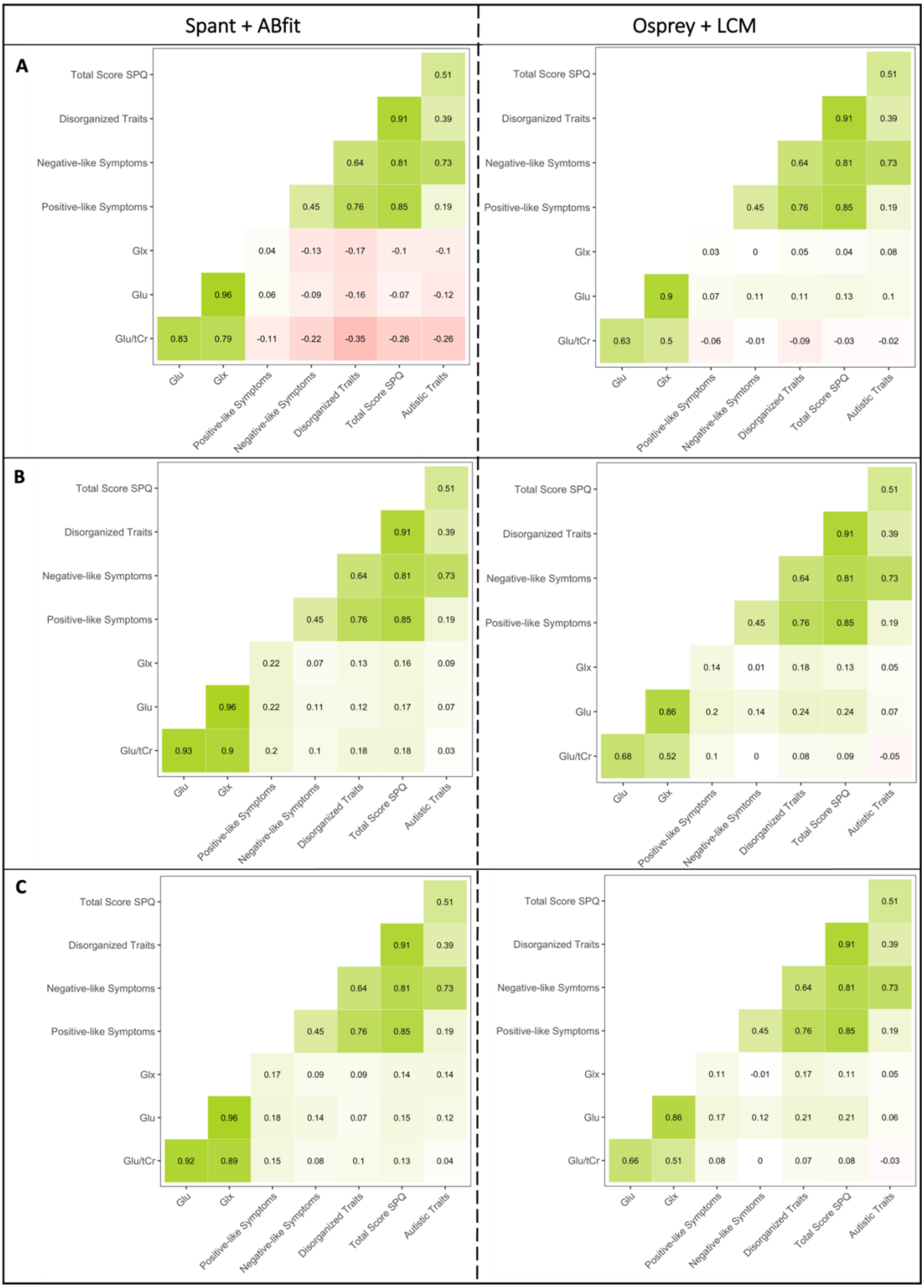

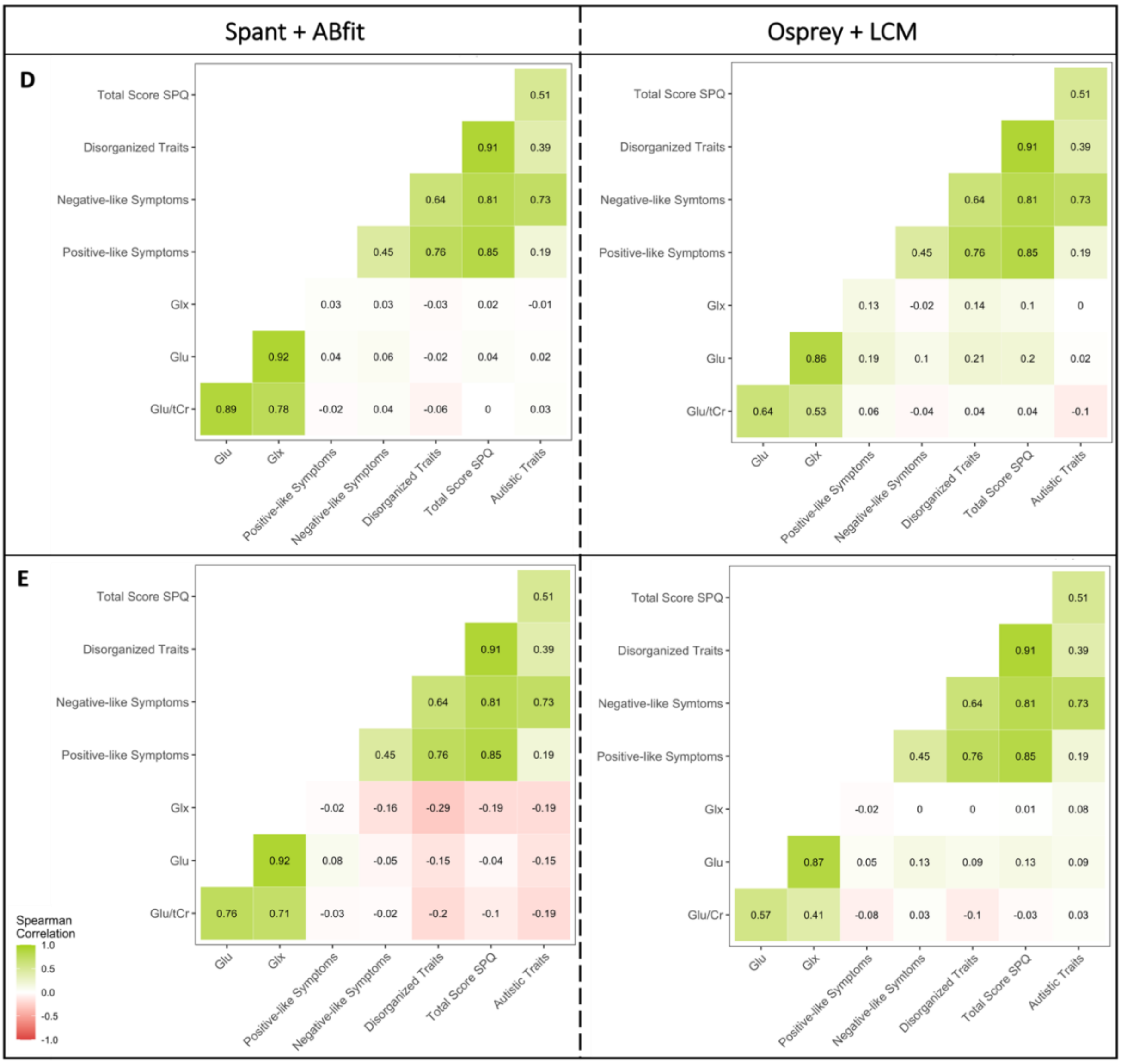

Note: Spearman correlations between Glu/tCr (Glu/Cr for Rowland and Kozhuharova), Glu, Glx, and the subclinical traits for the basis sets A)Rowland (9) *B) LCModel* (4) *C) Maddock* (2) *D) Reid* (3) *E) Kozhuharova* (5)*; the left side always shows the results for spant+ABfit and the right side for Osprey+LCM. The psychotic-like traits are separated into the subscores: positive-like symptoms, negative-like symptoms, and disorganized traits.*

Figure S5: Correlation of Glu/tCr, Glu and Glx with age by sex for spant+ABfit

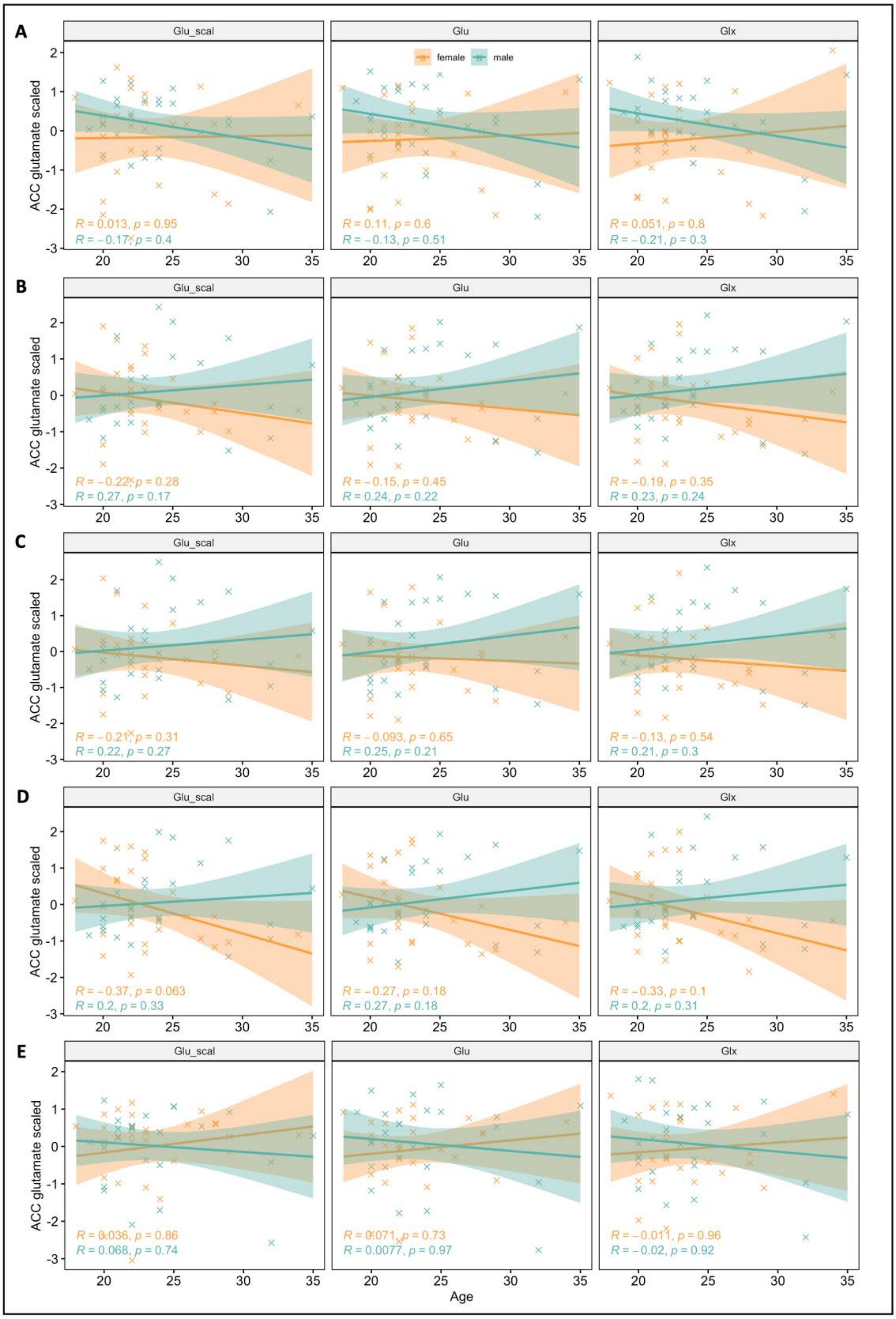

Note: Spearman correlations between Glu/tCr (Glu/Cr for Rowland and Kozhuharova), Glu, Glx, analysed with spant+ABfit, and the age by sex for A) Rowland (9) *B) LCModel* (4) *C) Maddock* (2) *D) Reid* (3) *E) Kozhuharova* (5)

Figure S6: Correlation of Glu/tCr, Glu and Glx with age by sex for Osprey+LCM

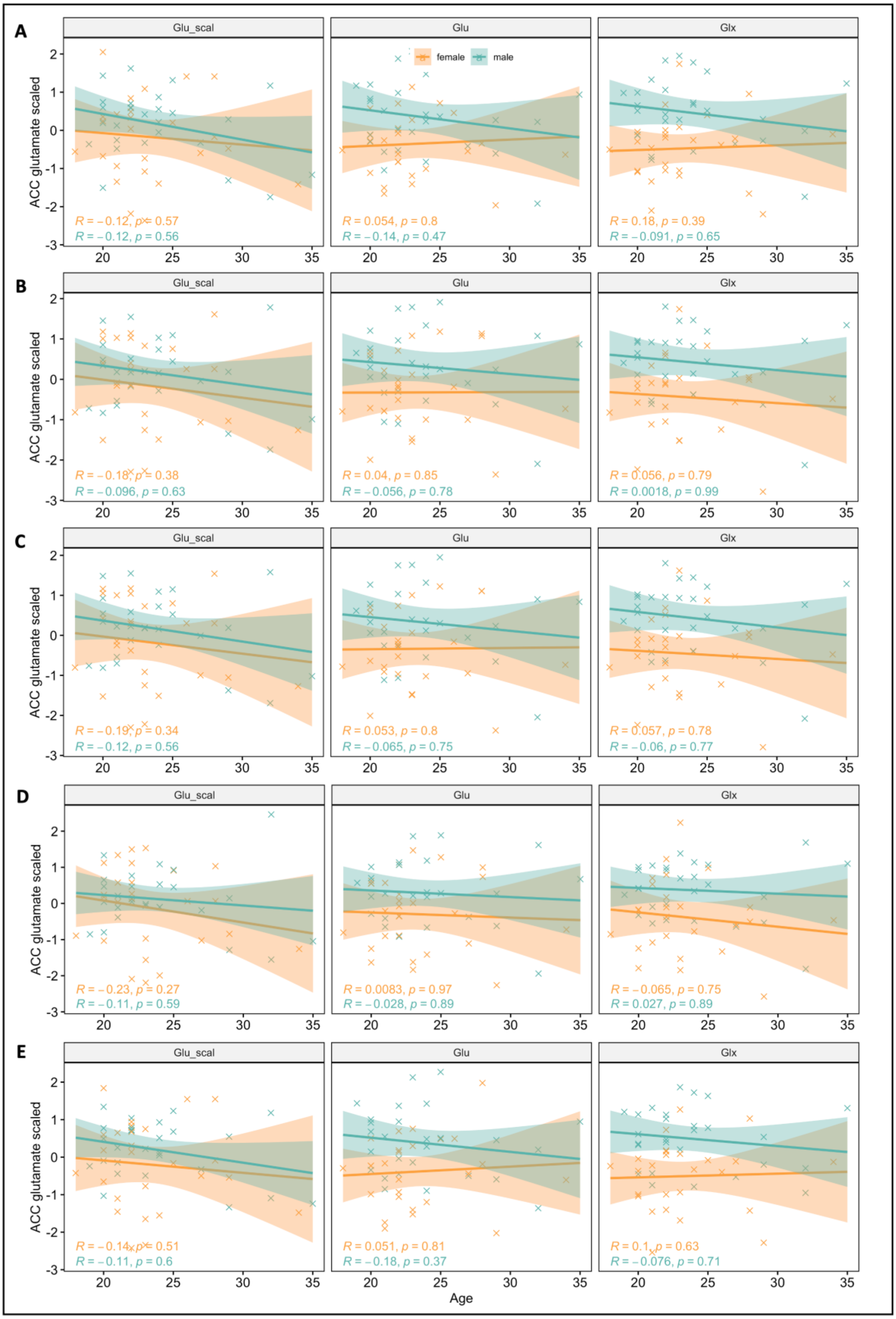

Note: Spearman correlations between Glu/tCr (Glu/Cr for Rowland and Kozhuharova), Glu, Glx, analysed with Osprey+LCM, and the age by sex for A) Rowland (9) *B) LCModel* (4) *C) Maddock* (2) *D) Reid* (3) *E) Kozhuharova* (5)

Figure S7: Group comparisons for Gln and Correlations between the metabolite concentrations and clinical scores

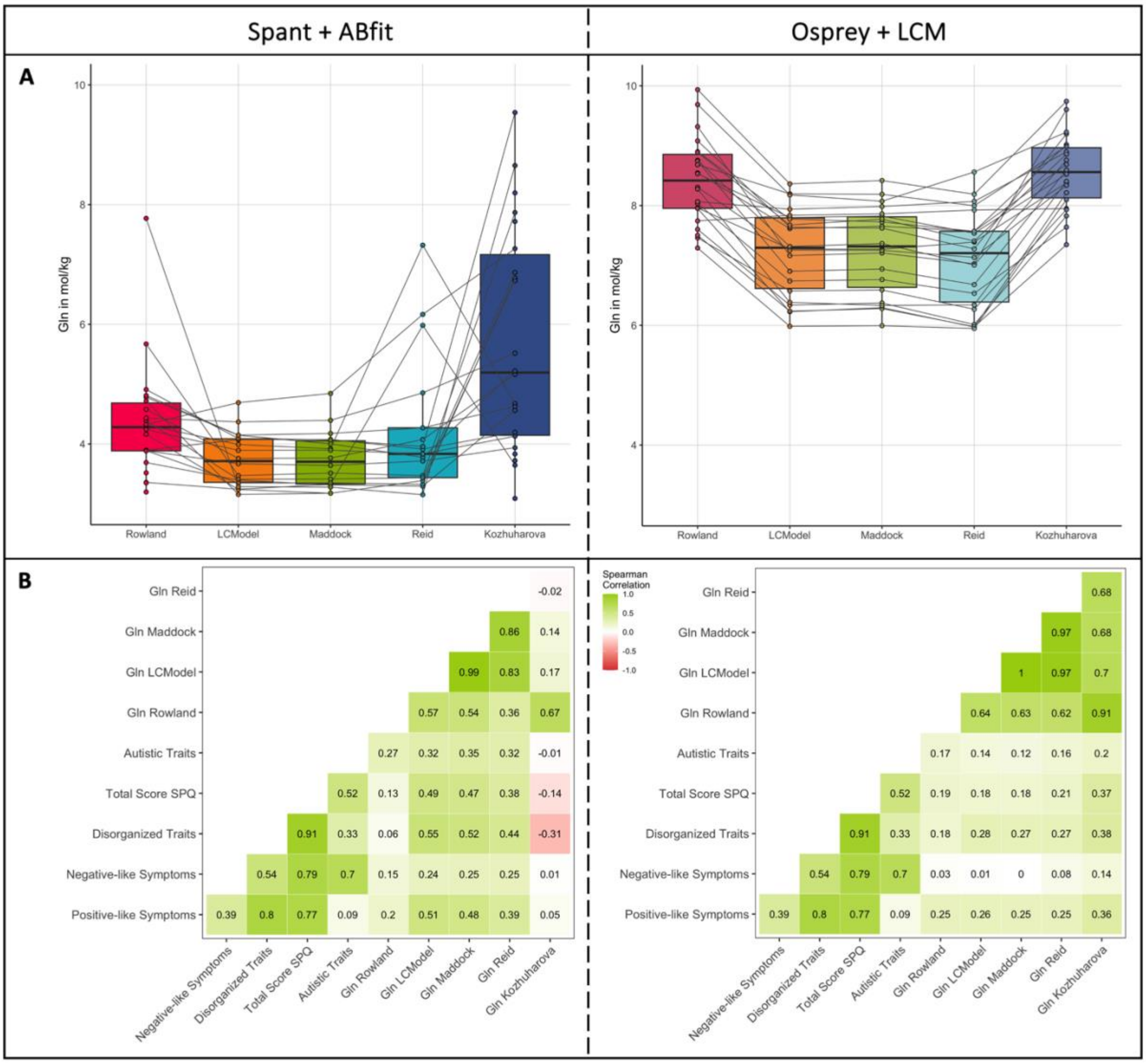

Note: A) Group comparisons between the basis sets Rowland (9)*, LCModel* (4)*, Maddock* (2)*, Reid* (3) *and Kozhuharova* (5) *for absolute Gln values; B) Spearman correlations between and the subclinical traits for the basis sets; the left side always shows the results for spant+ABfit and the right side for Osprey+LCM. The psychotic-like traits are separated into the subscores: positive-like symptoms, negative-like symptoms, and disorganized traits.*

Figure S8: Toolbox intercorrelations for Gln

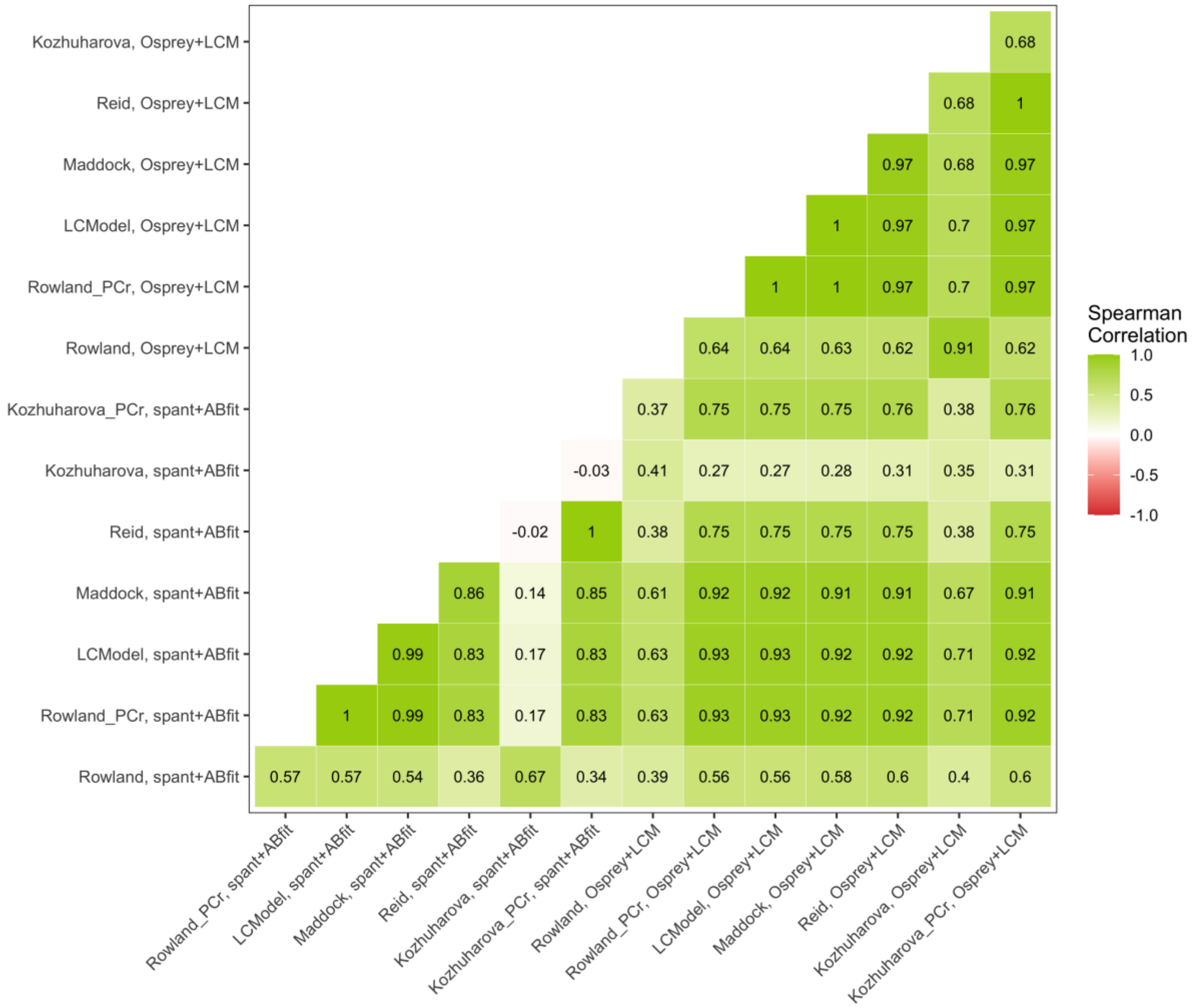

Note: Intercorrelations between the basis sets (Rowland (9)*, Rowland+PCr, LCModel* (4)*, Maddock* (2)*, Reid* (3)*, Kozhuharova* (5) *and Kozhuharova+PCr) and toolboxes (spant+ABfit; Osprey+LCM) for Gln.*

References:

1. Cheng H, Wang A, Newman S, Dydak U. An investigation of glutamate quantification with PRESS and MEGA-PRESS. NMR in Biomedicine. 2021;34(2):e4453.
2. Maddock RJ, Caton MD, Ragland JD. Estimating Glutamate and Glx from GABA-Optimized MEGA-PRESS: Off-Resonance but not Difference Spectra Values Correspond to PRESS Values. Psychiatry Res Neuroimaging. 2018 Sep 30;279:22–30.
3. Reid MA, Salibi N, White DM, Gawne TJ, Denney TS, Lahti AC. 7T Proton Magnetic Resonance

Spectroscopy of the Anterior Cingulate Cortex in First-Episode Schizophrenia. Schizophr Bull. 2019 Jan 1;45(1):180–9.

4. Provencher S. LCModel1 & LCMgui User's Manual. 2021 Feb 4; Available from: http://lcmodel.ca/pub/LCModel/manual/manual.pdf
5. Kozhuharova P, Diaconescu AO, Allen P. Reduced cortical GABA and glutamate in high schizotypy. Psychopharmacology. 2021 Sep;238(9):2459–70.
6. Leptourgos P, Bansal S, Dutterer J, Culbreth A, Powers A III, Suthaharan P, et al. Relating Glutamate, Conditioned, and Clinical Hallucinations via 1H-MR Spectroscopy. Schizophrenia Bulletin. 2022 Jul 1;48(4):912–20.
7. Ford TC, Nibbs R, Crewther DP. Glutamate/GABA+ ratio is associated with the psychosocial domain of autistic and schizotypal traits. Fatemi SH, editor. PLoS ONE. 2017 Jul 31;12(7):e0181961.
8. Shukla DK, Wijtenburg SA, Chen H, Chiappelli JJ, Kochunov P, Hong LE, et al. Anterior Cingulate Glutamate and GABA Associations on Functional Connectivity in Schizophrenia. Schizophrenia Bulletin. 2019 Apr 25;45(3):647–58.
9. Rowland LM, Kontson K, West J, Edden RA, Zhu H, Wijtenburg SA, et al. In Vivo Measurements of Glutamate, GABA, and NAAG in Schizophrenia. Schizophrenia Bulletin. 2013 Sep 1;39(5):1096–104.
10. Demler VF, Sterner EF, Wilson M, Zimmer C, Knolle F. Association between increased anterior cingulate glutamate and psychotic-like experiences, but not autistic traits in healthy volunteers. Sci Rep. 2023 Aug 7;13(1):12792.
11. Kassambara A. rstatix: Pipe-friendly framework for basic statistical tests [Internet]. 2022. Available from: https://CRAN.R-project.org/package=rstatix
12. Kassambara A. ggpubr: 'ggplot2' based publication ready plots [Internet]. 2022. Available from: https://CRAN.R-project.org/package=ggpubr
13. Akoglu H. User's guide to correlation coefficients. Turkish Journal of Emergency Medicine. 2018 Sep;18(3):91–3.
14. Marsman A, Mandl RCW, van den Heuvel MP, Boer VO, Wijnen JP, Klomp DWJ, et al. Glutamate changes in healthy young adulthood. Eur Neuropsychopharmacol. 2013 Nov;23(11):1484–90.

15. Hädel S, Wirth C, Rapp M, Gallinat J, Schubert F. Effects of age and sex on the concentrations of glutamate and glutamine in the human brain. J Magn Reson Imaging. 2013 Dec;38(6):1480–7.
16. Shimizu M, Suzuki Y, Yamada K, Ueki S, Watanabe M, Igarashi H, et al. Maturational decrease of glutamate in the human cerebral cortex from childhood to young adulthood: a 1H-MR spectroscopy study. Pediatr Res. 2017 Nov;82(5):749–52.
17. O'Gorman RL, Michels L, Edden RA, Murdoch JB, Martin E. In vivo detection of GABA and glutamate with MEGA-PRESS: Reproducibility and gender effects. Journal of Magnetic Resonance Imaging. 2011;33(5):1262–7.
